# Effects of Filling Rate and Resin Concentration on Pore Characteristics and Properties of Carbon Based Wood Ceramics

**DOI:** 10.3390/ma14092441

**Published:** 2021-05-08

**Authors:** Xiurong Guo, Qi Gao, Danfeng Du, Chaowei Sun

**Affiliations:** 1 College of Mechanical and Electrical Engineering, Northeast Forestry University, Harbin 150040, China; 15244682587@163.com; 2 School of Traffic and Transportation, Northeast Forestry University, Harbin 150040, China; ddf72@163.com (D.D.); suncw@nefu.edu.cn (C.S.)

**Keywords:** carbon based wood ceramics, resin concentration, filling rate, pore characteristics, properties, multiple regression model

## Abstract

As a kind of novel porous ceramics, wood ceramics can be used for filtration, friction, energy storage and electrode materials, etc. In current work, the carbon based wood ceramics (C WCMs) was prepared using pine wood powder and phenolic resin as starting materials. The effects of filling rate of wood powder and resin concentration on pore characteristics and properties of C WCMs were characterized and analyzed with different techniques. Furthermore, the association among porosity of C WCMs, filling rate of wood powder and resin concentration was explored with multiple regression model. The results showed that: increasing the resin concentration and the filling rate of wood powder can improve the mechanical properties of C WCMs, but reduce the porosity and air permeability; when resin concentration is more than 50%, a large amount of caking will appear in the C WCMs, causing internal defects; changing the filling rate under a certain resin concentration can obtain the C WCMs with better pore structure; the porosity of C WCMs has a good linear relationship with resin concentration and filling rate, under the condition that sintering process and the size of wood powder are determined.

## 1. Introduction

**Figure 1:**
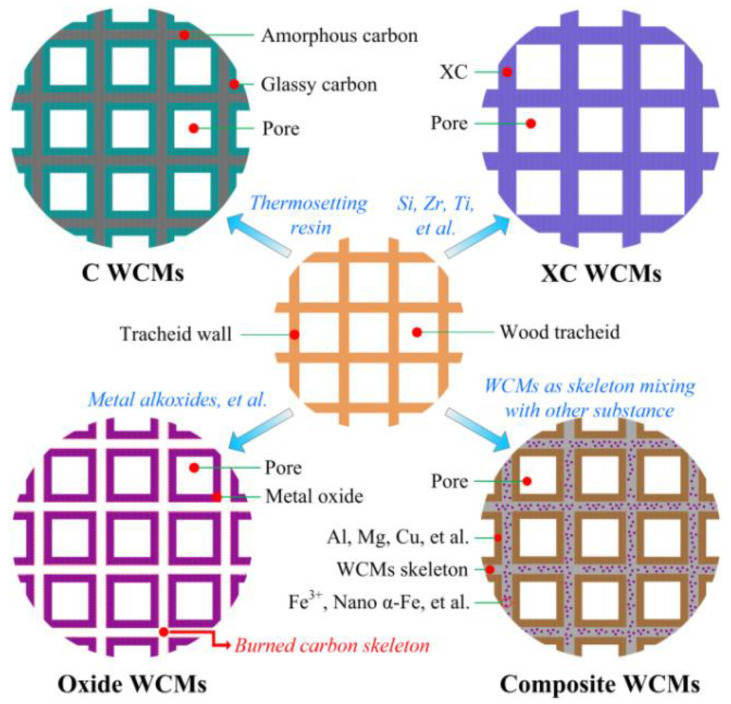
Schematics diagram of the microstructure of WCMs.

Porous ceramics are widely used in high temperature gas filtration [1,2], sewage purification [3,4], energy absorption [5,6], thermal insulation materials [7,8], ultrahigh temperature structural materials [9], electrolyte isolation [10], electromagnetic interference (EMI) shielding [11] and other energy and environmental technology fields. The key technology for producing novel porous ceramics with improved functions is to realize the interconnectivity and controllable preparation of complex microstructure units, which is also a challenge for the development of preparation technology of porous ceramics [12]. In the long development process of materials science, the preparation of new materials by imitating the characteristics of organisms in nature has broken through many technical difficulties, and then the biomimetic materials science was spawned, of which the wood ceramics is a typical example. Wood ceramics derived from natural wood or wood materials are porous ceramics, which completely retain the microscopic pore structure of natural wood [13,14]. According to the composition of substrate material, wood ceramics can be divided into 4 categories as shown in Figure 1: carbon based wood ceramics [15,16] (C WCMs), XC (SiC, ZrC, TiC et al.) based wood ceramics [17,18] (XC WCMs), oxide wood ceramics [19,20] (Oxide WCMs) and composite wood ceramics [21,22] (Composite WCMs). 

Compared with traditional porous ceramics, WCMs has the following advantages in pore regulation. Firstly, in terms of nature wood, the microscopic pore structure of wood is the result of natural selection, which makes it in a state of near perfect mechanical equilibrium [23] and the diversity of wood species and tissue structure make the change of microscopic pore structure of wood infinite [24]. In addition, in terms of the preparation process of WCMs, by changing the resin concentration, the filling rate of raw material, sintering process or using chemical and physical activation method, the microscopic pore structure of WCMs can also be regulated.

To the best of our knowledge, there are few studies on the regulation of the pore structure of WCMs. Pan J. et al. [25] utilized sugarcane bagasse as biological template high temperature sintering at 800 °C, 900 °C and 1100 °C to prepare C WCMs showing that, as the sintering temperature increasing, the total porosity of the C WCMs increases, but the specific surface area decreases due to the destruction of mesopores (2–50 nm) and micropores (<2 nm) in C WCMs. Sun D. et al. [26,27]. prepared C WCMs from liquefied wood and pine wood powder, and then the influence of chemical activation (KOH) and sintering process (sintering temperature, holding time and heating rate) on the mesopores, micropores and specific surface area of C WCMs were characterized, the experimental results of which showed that: the activation of KOH can significantly improve the microscopic pore structure of C WCMs; as the activation temperature increasing, the specific surface area of C WCMs increases first and then decreases, but the average pore size decreases first and then increases; as the sintering temperature increasing or the holding time prolonging, the average pore size of C WCMs tends to decrease first and then increase, but the heating rate has little effect on the average pore size of C WCMs. Zollfrank C. et al. [28] prepared Composite WCMs from natural pine and beech sample. The low molecular weight substances in the pine and beech samples were extracted in advance, and then some samples were chemically modified with MA (maleic acid anhydride). The experimental results showed that: wood species directly affect the microstructure of Composite WCMs; the Composite WCMs prepared from extracted samples has higher porosity; compared with untreated sample, after MA chemical modification, the ceramic yield of Composite WCMs is higher. In terms of the preparation of SiC WCMs, some researchers have improved the microscopic pore structure of SiC WCMs by physical activation of biocarbon templates. Herzog A. et al. [29] utilized nano SiO_2_ as silicon source to infiltrate biocarbon templates which were activated by CO_2_ at a high temperature of 900 °C, to prepare SiC WCMs, showing that, high temperature CO_2_ activation significantly increases the specific surface area of the biocarbon template. Manocha S. M. et al. [30] utilized pine wood as raw material, activating biocarbon template with high temperature steam (750 °C), for preparing SiC WCMs by sol-gel method, showing that, after high temperature steam activation, the average pore diameter of carbon template can reach 2.7 nm and the specific surface area can reach 710 m^2^/g. The improvement of the microstructure of biocarbon template ultimately improved the pore characteristics and properties of SiC WCMs. In addition, Wang M. et al. [31] utilized NaOH and NaClO_2_ to improve natural balsa wood and then the improved balsa wood was impregnated with polycarbosilane (PCS). After high temperature sintering, the SiC WCMs was prepared, showing that: proper removal of lignin and hemicellulose from wood by chemical methods can improve the impregnation process of PCS; the SiC WCMs sample prepared by the balsa wood samples which were improved by NaOH method and then impregnated with 40 wt.% PCS has a porosity as high as 61.03%.

The regulation of the microscopic pore structure of WCMs has a significantly effect on its practical application performance. Gao R. et al. [32] utilized corn straw mixed with natural porous diatomite as starting material to prepare improved C WCMs, the experimental results of which showed that the improved C WCMs has wider pore size range 1000–3000 nm, larger mean pore diameter 2382.7 nm and higher porosity 48.6%, and after regeneration, the removal efficiency of the improved C WCMs for tetracycline is above 90%. Yu X. et al. [33] utilized Ni(NO_3_)_2_ and K_2_CO_3_ to catalyze and activate waste poplar which was pre-impregnated with phenolic resin and assembled by lignin, then high temperature sintering at 800 °C, 1000 °C and 1200 °C, respectively, to prepare blocky C WCMs electrode, the experimental results of which showed that, the C WCMs prepared by K_2_CO_3_ activation and sintering at 1000 °C has better pore structure with specific surface area of 627.36 m^2^·g^−1^ and average pore diameter of 3.237 nm, and the better pore structure makes the blocky C WCMs electrode has a larger specific capacitances of 162.84 F·g^−1^ and a relatively small EIS (Electrochemical Impedance Spectroscopy) values of 0.432 Ω. Orihuela M. P. et al. [34] utilized Ayous, Pine, Iroko, Red oak and MDF (medium density fiberboard) as biological template to prepare SiC WCMs sample for purifying the DPM (diesel particulate matter ) from diesel boilers, showing that the permeability and purification efficiency of the sample are closely related to the microstructure parameters of SiC WCMs.

It can be seen that one of the key technical approach to improve the practical application performance of WCMs is to regulate its microscopic pore structure. However, at present, the study of pore regulation of WCMs is still at the initial exploration stage. Almost all research work is qualitative analysis and the quantitative description of the pore regulation law of WCMs has not been provided. In current work, C WCMs was prepared from pine wood powder and phenolic resin, and then the effects of resin concentration and filling rate of wood powder on pore characteristics and properties of C WCMs were analyzed experimentally. Moreover, in order to shorten the preparation period of C WCMs with specific porosity, the association among porosity of C WCMs, resin concentration and filling rate of wood powder was statistically analyzed utilizing multiple regression model, then the experience formula of which was provided.

## 2. Material and Methods

### 2.1. Sample Preparation

**Figure 2:**
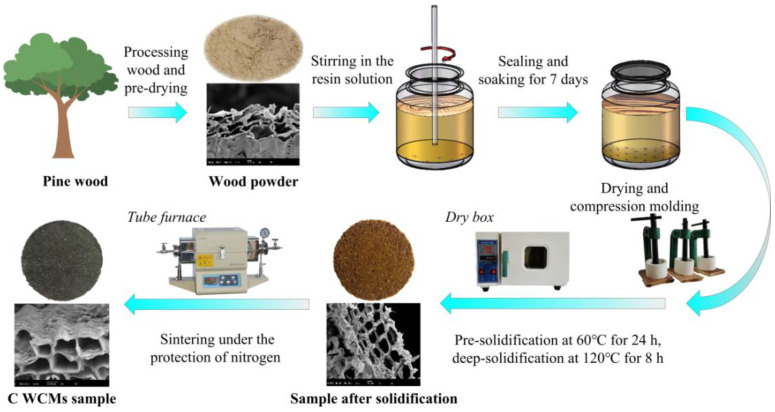
Preparation process route of the C WCMs sample.

The starting materials for preparing C WCMs include: self-made wood powder (20 mesh, red pine, Greater Khingan Range, China), phenolic resin (solid content 70.5%, viscosity 2.8 Pa·s; 2130; Zetian Chemical Co., Ltd.; Hengshui, China), ethanol (95% purity) and nitrogen (99.999% purity). The preparation process route is shown in Figure 2, the specific preparation process of which is as follows:

- Pry-drying: Dry the wood powder in an electrothermal blowing dry box (101-0; Zhetai Machinery Manufacturing Co., Ltd.; Shanghai, China) for 12 h at 110 °C.
- Soaking: A certain quality of wood powder was put into a self-made sealable tank soaking for 7 days under normal temperature and pressure conditions. The soaking solution made of ethanol and phenolic resin was in the sealable tank, the mass fractions of which were 20 wt.%, 30 wt.%, 40 wt.%, 50 wt.%, and 60 wt.%, respectively. Stir every 12 h to make the wood powder and soaking solution evenly mixed.
- Drying: Poured out the solution after soaking, and then placed the wood powder with different mass fraction of phenolic resin in the electrothermal blowing dry box drying for 12 h at 30 °C.
- Compression molding: Put a certain quality of wood powder impregnated with phenolic resin into a self-made molding device to press into a disc with thickness of 5 mm and diameter of 30 mm.
- Solidification: The molding device with wood powder was placed in the electrothermal blowing dry box, pre-solidification at 60 °C for 24 h, and then deep-solidification at 120 °C for 8 h to prepare the preforms of C WCMs.
- Sintering: The preforms were sintered into C WCMs in a tube furnace (TL1200; Boyuntong Instrument Technology Co., Ltd.; Nanjing, China). The heating process was as follows: room temperature −150 °C, 5 °C /min; 150–600 °C, 1 °C /min; 600–800 °C, 5 °C /min; 800 °C, keeping for 4 h. Nitrogen was continuously supplied as protective gas during the sintering process.

### 2.2. Characterization

#### 2.2.1. X-ray Diffraction

The phase composition of C WCMs was characterized by a X-ray diffractometer (XRD; XRD-6100; Shimadzu, Japan) with monochromator in the continuous mode in the range 2θ = 10–90°, using Cu Kα radiation, with a scanning rate of 1°·min^−1^ and time per step of 0.5 s. The crystallite size (d) of C WCMs was calculated from the full width at half-maximum (fwhm) of the diffraction peaks using Scherrer’s equation as follows:

(1) $$d=\frac{K\lambda }{fwhm\cdot cos\theta }$$

where *θ* = Bragg angle and *λ* = 0.15418 nm.

#### 2.2.2. Fourier Transform Infrared Spectroscopy

The fourier transform infrared spectrometer (FTIR; Spectrum 400; PerkinElmer, Waltham, MA, USA)as utilized to characterize the chemical composition of C WCMs, using diffuse reflection method to test the samples that were prepared by mixing crushed C WCMs and KBr with a mass ratio about 1:100. The number of scans was 80 and the resolution was set to 4 cm^−1^.

#### 2.2.3. Scanning Electron Microscopy

The scanning electron microscope (SEM; QUANTA 200; FEI, Valley City, ND, USA) utilized to characterize the microscopic pore morphology of C WCMs. The surface of the sample was carefully polished. After that, the sample was mounted on the aluminum holder with conductive adhesive and then sputtered with gold. Then the microscopic pore morphology of the samples was observed under the acceleration voltage of 13 kV.

#### 2.2.4. Porosity

Archimedes principle [35] was utilized to measure the porosity of C WCMs. The sample was boiled in boiling water for 1 h and then suspended in water to weigh. After that, took out the sample, wiped the water on the surface of the sample and then weighed it. Finally, the sample was placed in an electrothermal blowing dry box drying for 2 h at 110 °C. The porosity of the sample was calculated as follows:

(2) $$\mathrm{Porosity}=\frac{w_{\mathrm{wet}}-w_{\mathrm{dry}}}{w_{\mathrm{wet}}-w_{\mathrm{suspend}}}\cdot 100\%$$

where w_suspend_ represents the weight of the sample suspended in water; w_wet_ represents the weight of the sample after wiping the surface water; w_dry_ represents the weight of the sample after drying.

#### 2.2.5. Air Permeability

**Figure 3:**
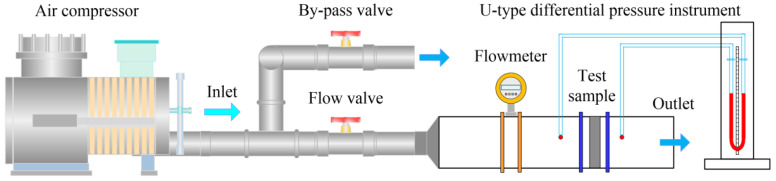
Schematic diagram of air permeability test of C WCMs samples.

Figure 3 illustrates the schematic diagram of air permeability test of C WCMs sample. The air compressor was utilized as the air power source. Set by-pass valve and flow valve, respectively, to control the air flow through C WCMs sample, where the air flow was measured by a vortex flowmeter. The pressure drop generated by air flowing across C WCMs sample was measured with a self-made U-type differential pressure instrument. The air permeability coefficients were obtained according to Darcy’s law and Forchheimer’s extension [36,37]:

(3) $$\frac{∆P}{L}=-\frac{\eta }{k_{1}}\cdot v-\frac{\rho }{k_{2}}\cdot v^{2}$$

where ΔP is the pressure drop (Pa) across the C WCMs sample; L is the thickness (m) of C WCMs sample; η is the dynamic viscosity (Pa·s); k_1_ is the Darcian permeability (m^2^); k_2_ is the inertial permeability (m); ρ is gas density (kg·m^−3^); v is gas flow velocity (m/s), v = Q/A; Q is gas flow (m^3^·s^−1^); A is the flow area (m^2^) across the C WCMs sample. For estimating the values of parameters k_1_ and k_2_, the second order polynomial with zero constant term was utilized to fit the experimental results of pressure drop and gas flow velocity.

#### 2.2.6. Mechanical Tests

According to the ASTM C 1499-19 testing standard, the mechanical tests of C WCMs samples were tested using the electro-mechanical universal testing machine (WDW-100; Kexin; Changchun, China). Adopting coaxial ring forcing method, the flexure strength ($\sigma_{f}$) was determined by measuring the breaking load (F) of the C WCMs sample, which was calculated by Equation (4). The compressive strength ($\sigma_{c}$) was determined by measuring the maximum crushing load (P) of the C WCMs sample, which was calculated by Equation (5).

(4) $$\sigma_{f}=\frac{3F}{2{\pi h}^{2}}\left[\left(1-v\right)\frac{\left(D_{S}^{2}-D_{L}^{2}\right)}{2D^{2}}+\left(1+v\right)\ln\frac{D_{S}}{D_{L}}\right]$$

(5) $$\sigma_{c}=\frac{4P}{{\pi D}^{2}}$$

where h is the thickness of the sample, mm; D_L_ is the diameter of the forcing ring, mm; D is the diameter of the sample, mm; v is the poisson’s ratio of the sample; D_s_ is the diameter of the support ring, mm.

#### 2.2.7. Ceramic Yield

The ceramic yield represents the mass ratio of C WCMs sample to the sample before high temperature sintering. The calculation method of ceramic yield is as follows:

(6) $$C_{y}=\frac{W_{2}}{W_{1}}\cdot 100\%$$

where W_1_ and W_2_ represent the mass of sample before and after sintering.

#### 2.2.8. Micro-Hardness Test

Polish the surface of C WCMs samples with diamond abrasive pastes. The HV-1000 micro-hardness tester was utilized to perform Vickers indentation test on the polished surface of the C WCMs sample, with indentation load of 1.96 N and duration time of 15 s. Each sample was tested at 9 different points, and the micro-hardness of the C WCMs sample was calculated by the following equation:

(7) $$\mathrm{HV}=1.8544\cdot \frac{G}{L^{2}}$$

where G is the indentation load, N; *L* is the diagonal size of the indentation, mm.

### 2.3. Statistical Analyses

Under the premise of determining the species of wood, the mesh number of wood powder and the sintering process, this study statistically investigated the effects of filling rate of wood powder and resin concentration of phenolic resin on the porosity of C WCMs, for quantizing the association among them. The multivariate regression model was utilized to display the influence of filling rate of wood powder and resin concentration on the porosity of C WCMs, as shown in the following expression:

Y=α_0_+β_1_X_1_+β_2_X_2_+ε (8)

where Y is the porosity of C WCMs; X_1_ is the filling rate of wood powder; X_2_ is the resin concentration of phenolic resin; α_0_ is the constant (intercept term) and ε is the residuals (error term). The ordinary least square (OLS) method was used to determine the regression coefficient of Equation (8). Generally, the expected value of error term is 0, namely, E(ε_i_) = 0. The variance inflation factor (VIF) values of all independent variables were analyzed in order to assess the multi-collinearity of the independent variables, where if the VIF value is not exceed 10 the multi-collinearity is not serious. Moreover, the Pearson correlation analysis was utilized to illustrate the significance of correlation between the two independent variables (X_1_, X_2_) and the dependent variable Y.

## 3. Results and Discussion

In this section, firstly, the changes in the phase and chemical composition of C WCMs were analyzed according to the XRD and FTIR characterization results. Then, the effects of resin concentration and filling rate of wood powder on the pore characteristics of C WCMs were investigated by the results of SEM characterization. Meanwhile, the effects of resin concentration and filling rate of wood powder on the porosity, air permeability, mechanical properties, ceramic yield and micro-hardness of C WCMs were also analyzed. Finally, in order to provide empirical guidance for the subsequent preparation of C WCMs, the association of porosity of C WCMs versus resin concentration and filling rate of wood powder were established utilizing multiple linear regression model.

### 3.1. Phase and Chemical Analysis

**Figure 4:**
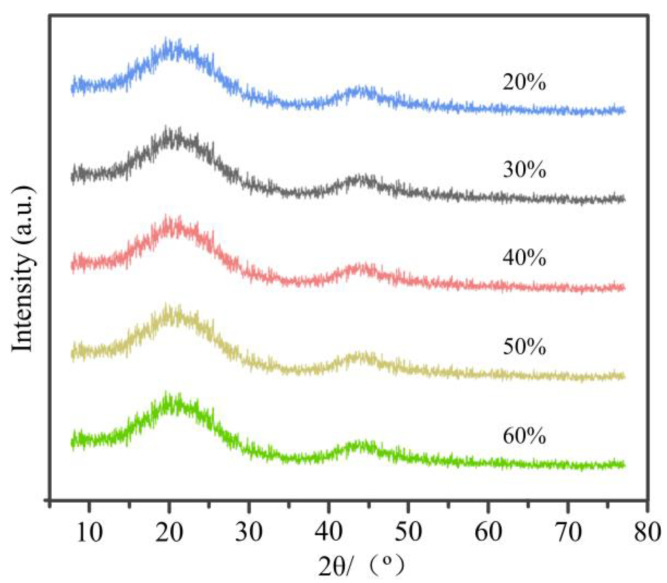
XRD patterns of C WCMs with different resin concentration sintering at 800 °C.

Figure 4 illustrates the XRD patterns of C WCMs with different resin concentration sintering at 800 °C. As shown from the figure that, there are two diffraction peaks similar to graphite in the XRD patterns, which are broad (002) diffraction peak and (10l) diffraction peak with weaker intensity, respectively. This indicates that the graphite microcrystalline in C WCMs is underdeveloped, disorder in arrangement and low in degree of orientation. Moreover, the typical diffraction peak of the linear molecule of cellulose from wood at 2θ ≈ 18.5° [38] has not been found. These results indicate that both wood and phenolic resin are all transformed into amorphous carbon during the high temperature sintering process. In fact, the amorphous carbon produced by carbonization of wood powder and the glassy carbon produced by carbonization of phenolic resin are both amorphous structure of carbon [39]. In addition, during the carbonization process, the hydroxyl functional groups of wood powder may react with the benzene ring of phenolic resin, causing the break and rearrangement of molecular chains of wood powder and phenolic resin, which also produces amorphous carbon. It can be concluded that the C WCMs made from phenolic resin and pine wood powder after high temperature sintering is a carbon-carbon composite material composed of amorphous carbon. It can also be seen from the figure that the resin concentration has almost no effect on the XRD patterns of C WCMs, which indicates that the resin concentration does not affect the phase composition of C WCMs. 

**Figure 5:**
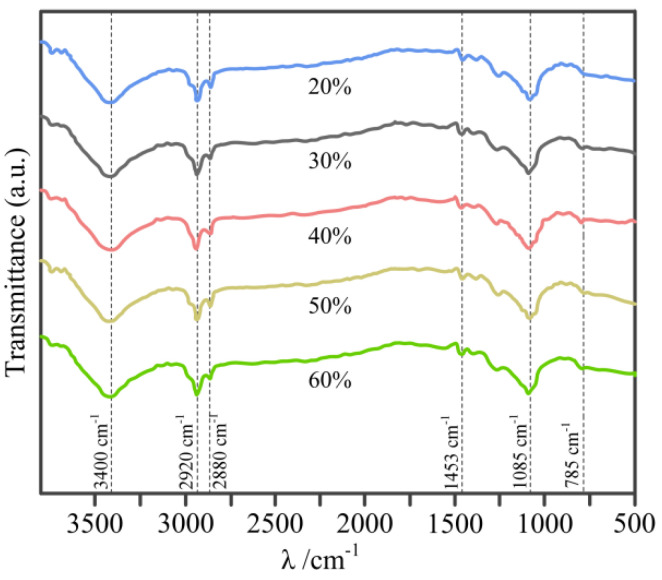
FTIR spectra of C WCMs with different resin concentration sintering at 800 °C.

Figure 5 depicts the FTIR spectra of C WCMs with different resin concentration sintering at 800 °C, where the absorption peak at 3430 cm^−1^ is O-H stretching vibration; the absorption peak at 2920 cm^−1^ is C-H asymmetric stretching vibration in aromatic series; the shoulder peak at 2880 cm^−1^ is C-H asymmetric stretching vibration in aliphatic series; the absorption peak at 1453 cm^−1^ is the C=C in-plane vibration peak in aromatic series; the absorption peak at 1085 cm^−1^ is the characteristic peak of C-O-C and the weak absorption peak at 785 cm^−1^ is C-H out-of-plane vibration in aromatic series. Whilst, the C=O absorption peak at 1710 cm^−1^ and 1740 cm^−1^ in nature wood were not found in C WCMs [40]. These results indicate that the C WCMs are carbon materials containing a small amount of O and H elements. Moreover, it can be seen from the figure that the resin concentration has no effect on the FTIR spectra of C WCMs, which indicates that the variation of resin concentration will not change the chemical composition of C WCMs.

In addition, the C WCMs with different filling rate of wood powder under constant resin concentration of 30% were also characterized by XRD and FTIR in current work, indicating that the filling rate of wood powder has no effect on the phase and chemical composition of C WCMs.

### 3.2. Pore Types of C WCMs

**Figure 6:**
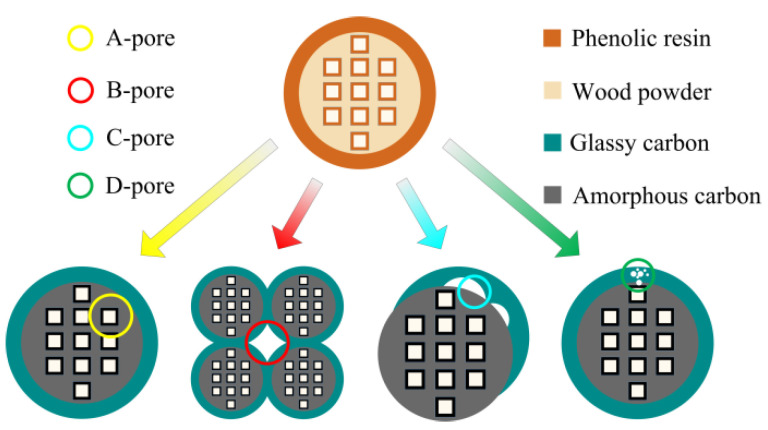
Schematic diagram of the pore formation principle of C WCMs.

Figure 6 shows the schematic diagram of the pore formation principle of C WCMs, which mainly contains four types of pores. As shown from the figure, A-pores are formed by duplicating the wood tracheids with regular shape, the internal surface of which is wrapped by glassy carbon, enhancing its mechanical properties. The pore size of A-pores is related to the resin concentration and the characteristics of wood (tree species, tree ages, sampling point). This is the typical characteristic and prominent advantage that distinguishes it from the microscopic morphology of traditional porous ceramics. Almost all tree species on the earth (hardwood or softwood), even herbs, all can be used as initial material of C WCMs, which increases the adjustable range of pore size and reduce the difficulty of pore regulation. B-pores are formed by interlaced connection of wood powder, and the joints of which are bonded by phenolic resin. The pore size of B-pores is related to the amount of phenolic resin and the filling rate of wood powder. C-pores are formed by the different shrinkage of wood powder and phenolic resin during high temperature sintering, the pore size of which is related to sintering process (maximum temperature, heating rate and holding time). In addition, there are nanoscale D-pores, which are formed by gasification of carbon material. The pore size of D-pores is related to sintering process [41] (maximum temperature, holding time).

### 3.3. Effect of Resin Concentration

**Figure 7:**
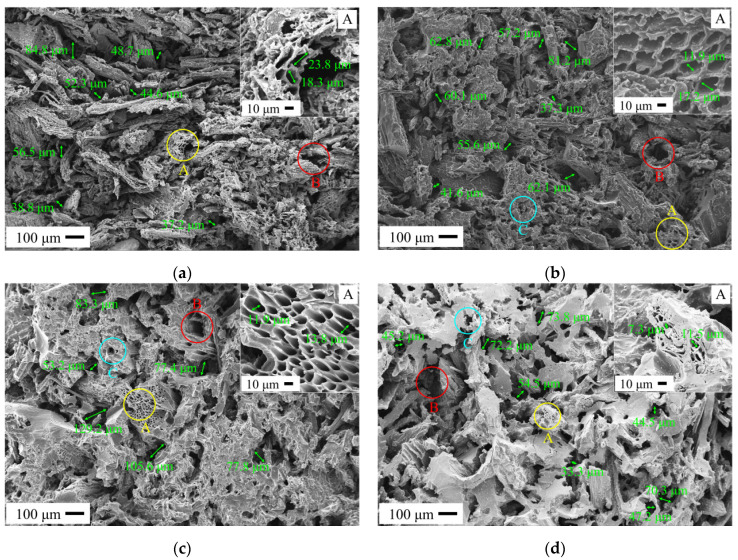

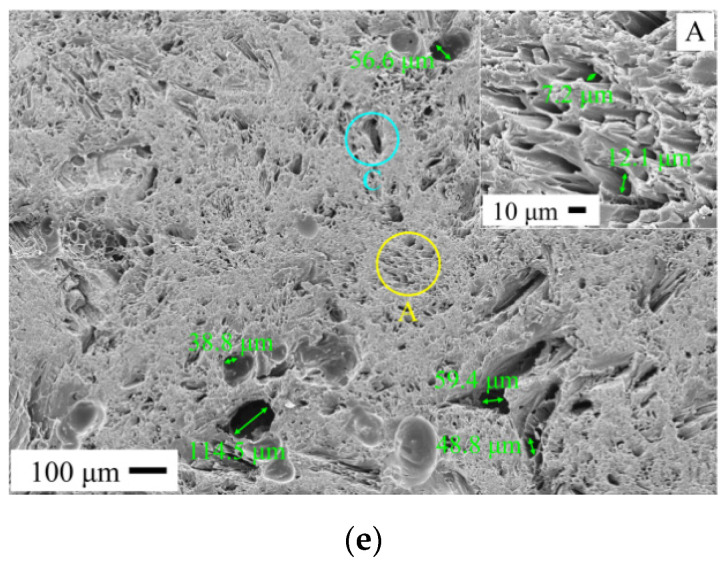
SEM micrographs of C WCMs with different resin concentration under a constant filling rate of wood powder of 37.5%: (**a**) resin concentration = 20%; (**b**) resin concentration = 30%; (**c**) resin concentration = 40%; (**d**) resin concentration = 50%; (**e**) resin concentration = 60%.

Figure 7 shows the SEM micrographs of C WCMs with different resin concentration under a constant filling rate of 37.5%, where the dark gray part represents amorphous carbon produced by carbonization of wood powder; the bright white part represents the glassy carbon produced by carbonization of phenolic resin; the black part represents the pores in C WCMs. As can be seen from the figure, with the resin concentration increasing, the area of the bright white part in SEM micrograph increases significantly. When the resin concentration is more than 50%, the dark gray part is hardly visible in the SEM micrograph, which indicates that the amorphous carbon is completely covered by glassy carbon. C WCMs not only retains the natural tracheid structure of wood to form A-pores, but also develops B-pores and C-pores with the bonding of phenolic resin, forming a heterogeneous 3D open pore structure with complex morphology. A-pores has regular shape and uniform pore size (5–30 μm), the internal channel of which is covered with a layer of glassy carbon. As the resin concentration increases, the thickness of the glassy carbon increases, causing the pore size of A-pores gradually decreases. In fact, there are a large amount of A-pores on each individual wood powder, but they cannot all be seen in the SEM micrograph, due to the difference of the cross section orientation of the sample and space distribution of wood powder. B-pores have no regular shape, the pore size of which ranging from 30 μm to 120 μm, which is related to the uniformity of the distribution of wood powder during the preparation process. As the resin concentration increases, the glassy carbon covering the surface of the wood powder increases. It can be observed from the figure that, when the resin concentration is more than 50%, the pores between wood powder are completely filled up with glassy carbon, forming caking in C WCMs, which makes the B-pores number reduce and the pore size shrink. In addition, there are also D-pores generated by the gasification of carbon materials exist in C WCMs, with pore size less than 20 nm [42], which cannot be observed in the SEM micrograph. As the resin concentration increases, the pore type, pore number and pore size of C WCMs gradually decrease.

**Figure 8:**
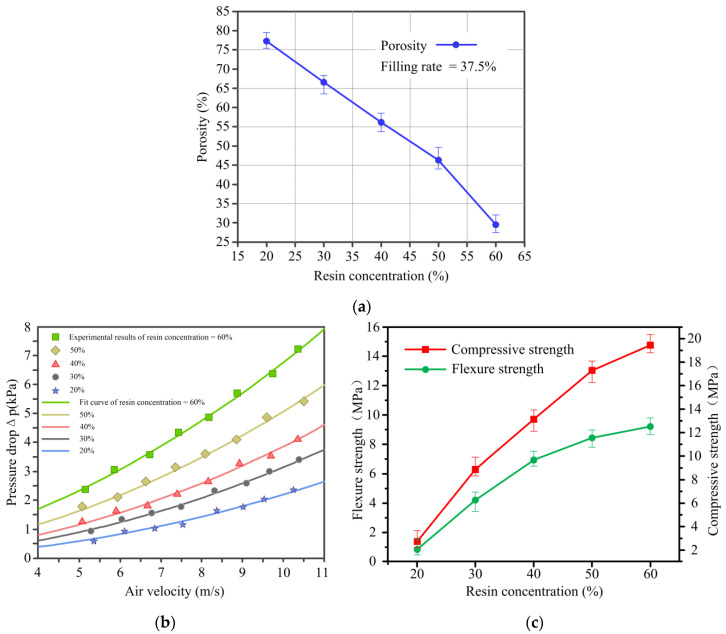
Experimental results of (**a**) porosity, (**b**) air permeability and (**c**) mechanical properties of C WCMs, with resin concentration of 20%, 30%, 40%, 50% and 60%, under a constant filling rate of wood powder of 37.5%.

Figure 8a shows the relationship between porosity of C WCMs and resin concentration under a constant filling rate of 37.5%. With resin concentration increasing, the porosity of C WCMs decreased from 77.2% to 28.5%. This is because, with the increasing of resin concentration, the number and size of pores in C WCMs decrease correspondingly, which is consistent with the analysis results of the SEM micrographs in Figure 7. The air permeability and mechanical properties of C WCMs are closely related to its porosity [24]. Figure 8b shows variation of pressure drop versus air velocity with different resin concentration under a constant filling rate of 37.5%. Utilizing the least square method to fit experimental results, the Darcian permeability (k_1_) and the inertial permeability (k_2_) of C WCMs sample with different resin concentration were calculated as shown in Table 1. The Darcian permeability of C WCMs prepared in current work is 2–3 orders of magnitude higher than that of SiC WCMs prepared by Orihuela M. P. et al. [34] research group, which indicates that the C WCMs made from wood powder has better air permeability. Compared with SiC WCMs prepared from nature wood, the C WCMs prepared in current work not only have A-pores that retains the natural tracheid structure of wood, but also generating B-pores, C-pores. Therefore, C WCMs has more diversified microscopic pore structure, which is the essential reason for its good air permeability. It can be found from the Figure 8b that the porosity of C WCMs is positively correlated with its air permeability. In addition, it can also be seen from Table 1 that the crystallite size of graphite sheets trends to decrease with the increasing of resin concentration, which has the same trends as the change in permeability constants of C WCMs. Figure 8c shows the relationship between mechanical properties and resin concentration under a constant filling rate of 37.5%. As the resin concentration increasing from 20% to 60%, the flexure strength of C WCMs increases from 0.91 MPa to 9.24 MPa, and the compressive strength increases from 2.43 MPa to 19.45 MPa. This is due to the mechanical properties of glassy carbon produced by phenolic resin are better than that of amorphous carbon, so as the resin concentration increasing, the mechanical properties of C WCMs are significantly improved. However, combined with the analysis results of SEM micrographs of C WCMs, when the resin concentration is more than 50%, the caking will form inside the C WCMs, resulting in uneven distribution of material density, which in turn causes internal defects of C WCMs. Therefore, the resin concentration should be controlled below 50%. Meanwhile, when the resin concentration is below 30%, C WCMs has higher porosity, but the mechanical properties are poor, and when the resin concentration is higher than 40%, the porosity of C WCMs is lower. In addition, it can also be found that the porosity of C WCMs is negatively correlated with its mechanical properties. It can be concluded that when the resin concentration is ranging from 30% to 40% under a constant filling rate of 37.5%, C WCMs has good mechanical properties, as well as high porosity and air permeability.

**Figure 9:**
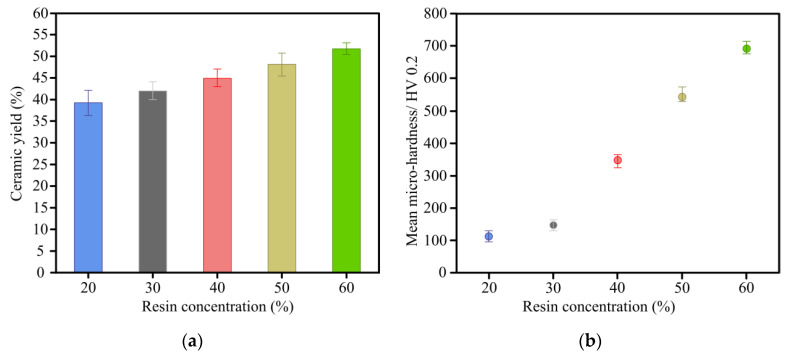
Experimental results of (**a**) ceramic yield and (**b**) micro-hardness of C WCMs with different resin concentration, under a constant filling rate of wood powder of 37.5%.

Figure 9a depicts the variation of ceramic yield versus resin concentration under a constant filling rate of 37.5%. Due to the removal of wood tar, wood vinegar and small molecular (CO_2_, CO, CH_4_ and H_2_) during sintering process, the weight of C WCMs sample is all decreased. However, with the resin concentration increasing from 20–60%, the ceramic yield of C WCMs increases from 39.5–51.8%, which is due to the higher carbon yield ratio of phenolic resin than that of wood powder under the same sintering process. Meanwhile, the content of glassy carbon produced by phenolic resin in C WCMs increases as the resin concentration increasing. The hardness of glassy carbon is higher than that of amorphous carbon produced by wood powder, so the micro-hardness of C WCMs increases significantly with the increasing of resin concentration, as shown in Figure 9b.

### 3.4. Effect of Filling Rate of Wood Powder

**Figure 10:**
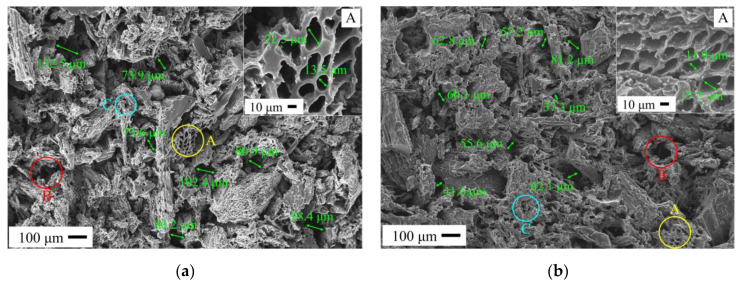

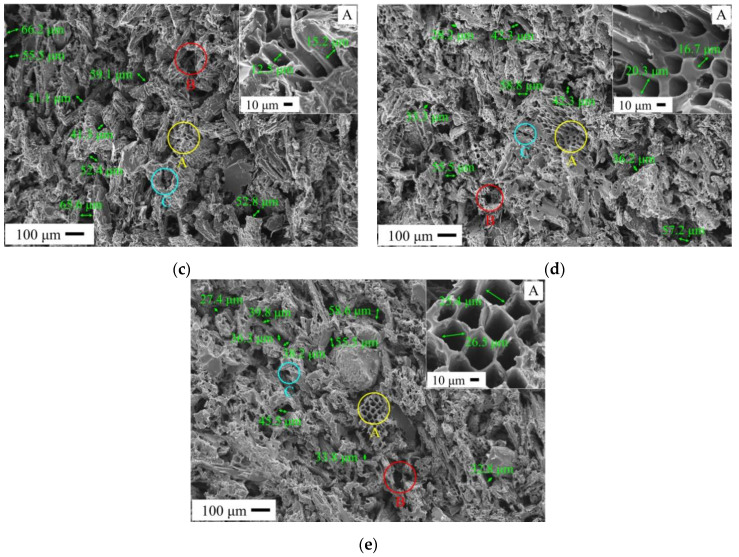
SEM micrographs of C WCMs with different filling rate of wood powder under constant resin concentration of 30%: (**a**) filling rate = 33.3%; (**b**) filling rate = 37.5%; (**c**) filling rate = 41.6%; (**d**) filling rate = 50%; (**e**) filling rate = 58.3%.

Figure 10 shows the SEM micrographs of C WCMs with different filling rate of wood powder under a constant resin concentration of 30%. When the filling rate of wood powder increasing from 33.3% to 58.3%, the area of dark gray and bright white in the cross section of C WCMs does not change obviously, indicating that the ratio of amorphous carbon to glassy carbon is almost the same. Meanwhile, with the filling rate of wood powder increasing, there is no large amount of caking in the cross section of C WCMs, which indicates that the amorphous carbon produced by wood powder is relatively uniformly distributed in C WCMs under the resin concentration of 30%. The internal channels of A-pores are covered with a layer of glassy carbon, which enhances the mechanical properties of amorphous carbon produced by wood powder. The pore size of A-pores is distributed in the range of 10–30 μm, which is no obvious correlation with the filling rate of wood powder. However, the pore size of B-pores is significantly reduced, from 73.9–132.5 μm (Figure 10a) to 27.4–58.6 μm (Figure 10e). This is because, with the filling rate of wood powder increasing, the extrusion between wood powders becomes more serious, which makes the pores formed by interlaced connection of wood powder reduce or even disappear. The pore characteristic of C-pores and D-pores are related to the sintering process, so changing the filling rate of wood powder will not affect them obviously.

**Figure 11:**
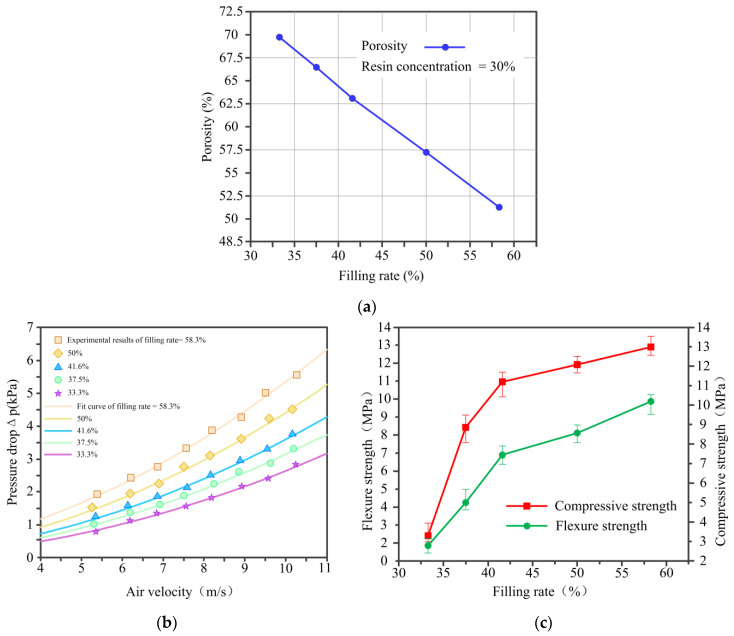
Experimental results of (**a**) porosity, (**b**) air permeability and (**c**) mechanical properties of C WCMs, with filling rate of wood powder of 33.3%, 37.5%, 41.6%, 50% and 58.3%, under a constant resin concentration of 30%.

Figure 11a shows the relationship between porosity of C WCMs and filling rate of wood powder under a constant resin concentration of 30%. As the filling rate of wood powder increasing from 33.3% to 58.3%, the porosity of C WCMs decrease from 69.8% to 51.3%, showing a linear change. Figure 11b shows the variation of pressure drop versus air velocity with different filling rate of wood powder under a constant resin concentration of 30%. The Darcian permeability (k_1_) and the inertial permeability (k_2_) of C WCMs with different filling rate of wood powder were calculated as shown in Table 2, which is positively correlated with the porosity of C WCMs. Moreover, it can also be seen from Table 2 that the crystallite size of graphite sheets is not significantly related to the filling rate and permeability constants. It can be concluded that the air permeability of C WCMs is determined by its microscopic pore structure. Figure 11c shows the relationship between mechanical properties and filling rate of wood powder under a constant resin concentration of 30%. With the filling rate of wood powder increasing from 33.3% to 58.3%, the flexure strength of C WCMs increases from 1.89 MPa to 9.85 MPa and the compressive strength increases from 3.26 MPa to 12.98 MPa. There are two reasons for this result. Firstly, the tighter the wood powder is squeezed, the resin attached to the surface of the wood powder will penetrate more into the tracheid cavity of wood powder, making the mechanical properties of C WCMs strengthen. Moreover, the squeezing of wood powder will make the amorphous carbon and glassy carbon bond more tightly at the interface, increasing the bonding strength of the two carbon materials, which finally enhances the mechanical properties of C WCMs. It can be found that when the filling rate is greater than 41.6% the increment of mechanical properties of C WCMs decreasing significantly. Meanwhile, when the filling rate is lower than 37.5%, the mechanical properties of C WCMs are poor. It can be concluded that when the filling rate is ranging from 37.5% to 41.6% under a constant resin concentration of 30%, C WCMs has good mechanical properties, as well as high porosity and air permeability.

**Figure 12:**
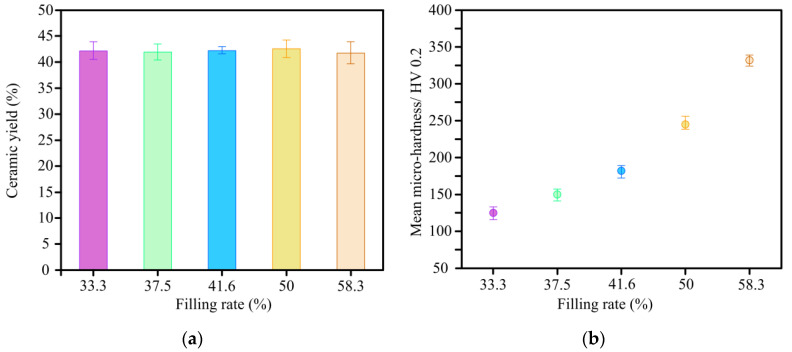
Experimental results of (**a**) ceramic yield and (**b**) micro-hardness of C WCMs with different filling rate of wood powder, under a constant resin concentration of 30%.

Figure 12a depicts the variation of ceramic yield versus filling rate of wood powder under a constant resin concentration of 30%. It can be seen that the increasing of filling rate of wood powder has almost no effect on the ceramic yield of C WCMs. This is because the concentration of phenolic resin is constant, that is, the mass ratio of phenolic resin to wood powder is the same for all samples, so the mass loss ratio of samples with different filling rate is the same after high temperature sintering. Even so, it can be seen from Figure 12b that the micro-hardness of C WCMs increases with the increasing of the filling rate, but not as drastic as the change in hardness caused by resin concentration. This is because with the filling rate increasing, the wood powder is squeezed more tightly, which allows more glassy carbon to be bound on the tracheid wall surface and the glassy carbon is more evenly distributed.

### 3.5. Statistical Analysis Results of Porosity

Through Pearson correlation analysis, the resin concentration X_2_ shows a very high negative correlation (Pearson correlation coefficient = −0.909) with the porosity Y of C WCMs, at a 1% level of significance. Meanwhile, the filling rate X_1_ also shows negative correlation (Pearson correlation coefficient = −0.407) with the porosity Y of C WCMs, at a 5% level of significance. The results indicate that, as the concentration of phenolic resin and the filling rate of wood powder increases, the porosity of C WCMs decreases and the resin concentration has a more significant effect on porosity. Moreover, the Pearson correlation coefficient among the independent variables (X_1_, X_2_) were almost 0, indicating that there is no multi-collinearity problem in regression analysis.

**Figure 13:**
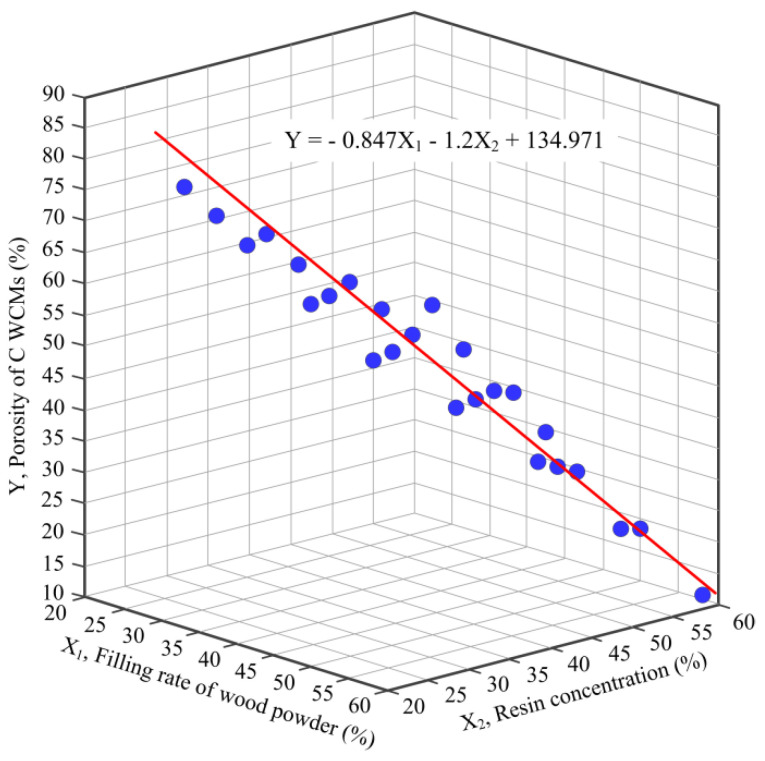
The association among the porosity of C WCMs, filling rate of wood powder and resin concentration, under the condition that the species of wood, the size of wood powder and the sintering process are determined.

Table 3 illustrates the regression result. The estimated coefficients of resin concentration (X_2_) and filling rate of wood powder (X_1_), obtained by multiple regression analysis are −1.2 and −0.847, respectively, which are all negatively significant at a 1% level and the regression curve is shown in Figure 13. The F-value was also significant at a 1% level and the adj.R^2^ was 0.99 indicating that the regression model fits well with experimental data and the model is acceptable. The VIF (variance inflation factor) for estimated coefficients of independent variables are all less than 10, indicating that the multi-collinearity problem is not significant.

## 4. Conclusions

In current work, C WCMs was prepared with phenolic resin and wood powder as starting materials, and the preparation process of C WCMs was obtained. The phase and chemical composition of C WCMs were characterized by XRD and FTIR. The effects of resin concentration and filling rate of wood powder on the microscopic pore structure, porosity, air permeability, mechanical properties and ceramic yield of C WCMs were analyzed with different techniques. Finally, the relationship between the porosity of C WCMs and resin concentration and filling rate of wood powder was determined utilizing multiple linear regression model. Following conclusions were reached:

1. C WCMs made from phenolic resin and pine wood powder is a carbon-carbon composite material, which is mainly composed of amorphous carbon and contains a small amount of O and H element. The resin concentration and filling rate of wood powder have no significant effect on the phase and chemical composition of C WCMs.
2. C WCMs not only completely retains the natural tracheid structure of wood to generate A-pores, but also develops B-pores, C-pores and D-pores under the bonding of phenolic resin, forming a three-dimensional heterogeneous net open pore structure with complex microscopic morphology.
3. Increasing the resin concentration and the filling rate of wood powder can improve the mechanical properties of C WCMs, but reduce the porosity and air permeability of C WCMs. When resin concentration is more than 50%, a large amount of caking will appear in the C WCMs, which not only reduce the type and quantity of pores of C WCMs, but also causes internal defects in the C WCMs. Whilst, increasing the filling rate of wood powder will significantly reduce the quantity and pore size of B-pores, but will not reduce the pore types of C WCMs.
4. Under the condition that sintering process and the size of wood powder are determined, the porosity (Y) of C WCMs has a linear correspondence with the filling rate (X_1_) of wood powder and resin concentration (X_2_), that is Y=−0.847X_1_−1.2X_2_+134.971. The resin concentration has greater impact on the porosity of C WCMs, but changing the filling rate of wood powder under a certain resin concentration can obtain the C WCMs with better pore structure.

## 5. Patents

Gao Q, Guo XR, Du DF. An experimental device for measuring the pressure drop of the filter sample made of wood fiber ceramic, ZL.2020 2 0001765.8, 2020 (In Chinese).

## Figures and Tables

**Table 1:** Permeability constants and crystallite size of C WCMs sample with different resin concentration under a constant filling rate of 37.5%.

Concentration (%)	Darcian Permeability k_1_ (×10^−10^ m^2^)	Inertial Permeability k_2_ (×10^−4^ m)	Crystallite Size d (nm)
20	45.5 ± 3.2	2.53 ± 0.7	0.3926
30	16.3 ± 1.5	1.92 ± 1.1	0.3883
40	9.48 ± 1.3	1.68 ± 0.5	0.3822
50	6.78 ± 0.8	1.45 ± 0.1	0.3764
60	4.67 ± 0.4	1.23 ± 0.2	0.3725

**Table 2:** Permeability constants and crystallite size of C WCMs sample with different filling rate under a constant resin concentration of 30%.

Filling Rate (%)	Darcian Permeability k_1_ (×10^−10^ m^2^)	Inertial Permeability k_2_ (×10^−4^ m)	Crystallite Size d (nm)
33.3	25.1 ± 2.1	2.21 ± 1.1	0.3875
37.5	16.3 ± 1.4	1.92 ± 0.9	0.3883
41.6	11.4 ± 1.2	1.75 ± 0.3	0.3881
50	8.21 ± 0.9	1.47 ± 0.1	0.3871
58.3	5.66 ± 0.6	1.28 ± 0.1	0.3892

**Table 3:** Result of regression.

Y=α_0_+β_1_X_1_+β_2_X_2_+ε			
Variables	Coef.	t-stat	VIF
Intercept	134.971	61.764	-
β_1_	−0.847	−20.037	1.0
β_2_	−1.2	−44.74	1.0
adj.R^2^	99.0%	-	-
F-value	1201	-	-
*p*-value	<0.001	-	-

## Data Availability

Data can be made available upon request.
